# Experimental Study of Mechanical Properties and Theoretical Models for Recycled Fine and Coarse Aggregate Concrete with Steel Fibers

**DOI:** 10.3390/ma17122933

**Published:** 2024-06-15

**Authors:** Cai Wu, Yan Shi, Jiale Xu, Mingxing Luo, Yani Lu, Daopei Zhu

**Affiliations:** 1 School of Civil Engineering, Hubei Engineering University, Xiaogan 432000, China; 2 Changjiang River Scientific Research Institute, Changjiang Water Resources Commission, Wuhan 430010, China; 3 Department of Civil Engineering, Kyushu University, Fukuoka 819-0395, Japan; 4 School of Civil and Surveying & Mapping Engineering, Jiangxi University of Science and Technology, Ganzhou 341000, China

**Keywords:** recycled aggregate, steel fiber, shrinkage performance, constitutive relationship

## Abstract

With diminishing natural aggregate resources and increasing environmental protection efforts, the use of recycled fine aggregate is a more sustainable approach, although challenges persist in achieving comparable mechanical properties. Exploration into the incorporation of steel fibers with recycled aggregate has led to the development of steel-fiber-reinforced recycled aggregate concrete. This study investigates the shrinkage performance and compressive constitutive relationship of steel fiber recycled concrete with different steel fibers and recycled aggregate dosages. Initially, based on different replacement rates of recycled coarse aggregate and different volume contents of steel fiber, experimental results demonstrate that as the replacement rate of recycled coarse aggregate increases, shrinkage also increases, while the addition of steel fiber can mitigate this effect. An empirical shrinkage model for steel fiber recycled concrete under natural curing conditions is also proposed. Subsequently, based on the uniaxial compression test, findings indicate that with an increasing replacement rate of recycled fine aggregate, the peak stress and elastic modulus of concrete decrease, accompanied by an increase in peak strain, and the addition of steel fiber limits concrete crack development and enhances its brittleness while the peak stress and strain of recycled fine aggregate concrete are enhanced. However, the steel fiber volume percentage has a negligible effect on the elastic modulus. A constitutive relationship for concrete considering the effects of recycled fine aggregate and steel fiber is also proposed. This finding provides foundational support for the influence patterns of steel fiber dosage and recycled aggregate ratio on the mechanical properties of steel fiber recycled concrete.

## 1. Introduction

With the advancement of urbanization, there has been a rapid increase in infrastructure development, exacerbating the demand for construction materials. Concurrently, numerous new projects are in the planning stages, resulting in a significant amount of construction waste through demolitions, renovations, and expansions [1]. The accumulation of such waste necessitates innovative solutions for management and recycling. Under these circumstances, the recycling and utilization of abandoned concrete, such as recycled concrete aggregates, have become focal points of research [2,3,4]. However, recycled concrete aggregates have different properties than traditional concrete due to initial defects. Adding steel fibers to recycled concrete can effectively control crack formation and compensate for its lower strength. However, the mechanical properties of recycled steel fiber concrete are still not quite clear.

Concrete shrinkage poses a significant challenge in engineering applications; it can lead to internal tensile stresses and the potential for shrinkage cracks [5], result in reduced structural load-bearing capacity, and, in severe cases, lead to engineering accidents that jeopardize both personal and property safety. Before studying the stress-strain behavior, understanding the shrinkage properties is quite important. Numerous factors influence the shrinkage performance of recycled concrete, including design methods, mix proportions, recycled aggregates, admixtures, and curing conditions. Research has shown that both the traditional replacement method and the equal-volume mortar method (EMV method) result in greater shrinkage compared to ordinary concrete [6]. Studies have demonstrated that the higher the quality of recycled aggregates, the more effectively they can inhibit the shrinkage of recycled concrete, even surpassing that of ordinary concrete [7]. Despite their minimal dosage, admixtures play a crucial role in enhancing concrete performance. It has been discovered that incorporating fly ash into recycled concrete effectively suppresses shrinkage by improving microstructure, reducing water demand, lowering hydration heat, and enhancing the interfacial transition zone [8]. However, it is not as big as better, so determining the optimal dosage for achieving the desired shrinkage reduction is quite important [9]. The addition of a small quantity of expansive agent can effectively minimize the shrinkage of recycled coarse aggregate concrete [10,11], making it comparable to ordinary concrete with 50% recycled content. Water-reducing agents contribute to the improvement of concrete’s pore structure, thereby mitigating the shrinkage performance of recycled concrete to a certain extent. Effective curing plays a crucial role in achieving concrete’s design requirements, and different curing methods such as water curing, covering with wet burlap or fabric, spray curing, plastic sheeting, chemical curing compounds, etc. can impact the shrinkage performance of concrete [12]. 

The study of modern fiber-reinforced concrete dates back over a century, starting in the early 20th century. The addition of fibers is primarily aimed at improving the brittle characteristics of concrete. However, different fiber types exhibit significant variations in performance, resulting in varying effects on concrete properties. The main types of fibers currently used include polypropylene fibers and steel fibers, while other fibers such as basalt fibers, carbon fibers, and eco-friendly fibers are also being explored. Previous research has demonstrated the effectiveness of incorporating polypropylene fibers in concrete to mitigate early-age shrinkage [13]. At doses below the optimal level, a higher dosage yields a more noticeable reduction in concrete shrinkage. However, surpassing the optimal dosage does not significantly enhance the inhibitory effect and may even impact the workability of concrete. Steel fibers are the most commonly used type of fiber in fiber-reinforced concrete, available in various forms such as hooked-end and milling-shape. Studies have demonstrated that different forms of steel fibers can effectively mitigate concrete shrinkage. Moreover, within a specific range of volume dosage, increasing the volume dosage of steel fibers leads to a more pronounced inhibitory effect on concrete shrinkage [14]. Wu et al. [15] found that the optimal dosage of steel fibers is around 1.5%. When steel fibers are added to concrete with a recycled coarse aggregate replacement ratio lower than 40%, similar strength and shrinkage levels to ordinary concrete can be achieved. However, excessive or inadequate dosage can diminish the effectiveness of shrinkage inhibition. The optimal dosage of steel fibers in concrete to achieve the best inhibition of shrinkage can vary depending on several factors, such as the type of steel fibers, concrete mix design, environmental conditions, and desired performance characteristics.

On the other hand, the uniaxial constitutive relationships of steel-fiber-reinforced recycled coarse aggregate concrete have been extensively studied. Folino et al. [16] demonstrated the similarity in constitutive relationships for recycled aggregate concrete at different coarse aggregate replacement rates of 50% and 100%. Gao et al. [17] developed a constitutive model for steel fiber-reinforced recycled concrete; considering the impact of recycled aggregate replacement rates of 30%, 50%, and 100% and the steel fiber volume fraction. While research findings on recycled coarse aggregate concrete are well documented in the literature, studies pertaining to recycled fine aggregate concrete remain relatively limited. Existing studies on recycled fine aggregate concrete have mainly focused on aspects such as its durability. For instance, Xiao et al. [18] and Bamigboye et al. [19] investigated the durability of recycled fine aggregate concrete, and the results showed that although the durability of recycled fine aggregate is slightly lower than that of ordinary concrete, it can still be used in concrete construction, making it practicable for engineering applications. 

In addition to the studies mentioned above, it can be concluded that existing theoretical models often do not fully account for the complex interactions in RAC with steel fibers. Current research has not systematically studied the impact of these two factors comprehensively. There is a need to develop predictive models that can accurately simulate mechanical behavior. The mechanical constitutive model and shrinkage model of recycled concrete are influenced by various factors. To fill this gap, taking into consideration that steel fibers can not only inhibit concrete shrinkage but also enhance the compressive strength of recycled concrete, this study first investigates the influence of different replacement rates (0%, 50%, 100%) of recycled coarse aggregates and varying volumes (0%, 0.5%, 1.0%, and 1.5%) of steel fibers on the shrinkage performance of recycled concrete. Additionally, a comparative analysis is conducted to evaluate the shrinkage performance of recycled concrete in comparison with that of ordinary concrete. Then, the effect of recycled fine aggregate replacement rate and steel fiber volume percentage on the mechanical performance of the steel-fiber-reinforced recycled fine aggregate concrete is also investigated. Steel fibers are incorporated into recycled fine aggregate concrete to improve its mechanical properties, and by varying the replacement ratio of recycled fine aggregate and the volume fraction of steel fibers, the compressive behavior of the concrete under axial loading is investigated. A stress-strain constitutive model is developed for steel fiber-reinforced recycled fine aggregate concrete. This study provides theoretical references for the application of recycled fine aggregate steel fiber concrete.

## 2. Experimental Design

### 2.1. Materials

#### 2.1.1. Shrinkage Performance Test

The experiment utilized PO.42.5R Portland cement from the “Hailuo” brand. The fine aggregate consisted of river sand with a fineness modulus of 2.38. Natural coarse aggregate consisted of granite crushed stone ranging in size from 5 mm to 25 mm. The recycled coarse aggregate was derived from laboratory waste blocks (C30), crushed, and sieved to match the particle size of the natural aggregate. After testing based on the Chinese Standard JGJ 52-2006 [20], the water absorption rate of the natural aggregate was 0.48%, with a crushing index of 7.71%. The recycled coarse aggregate exhibited a water absorption rate of 3.56%, along with a crushing index of 14.7%.

**Figure 1:**
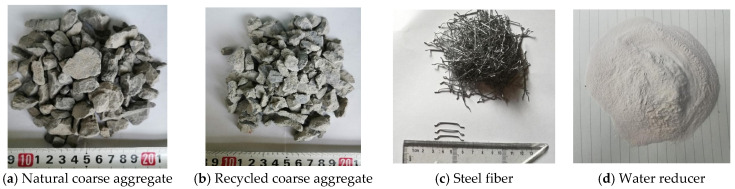
Photos of part materials.

The experiment employed end-hooked steel fibers produced by Jiangsu Subot Company (Nanjing, China). These fibers had a diameter of 0.52 mm, a length of 30 mm, and a tensile strength of 1060 MPa. The water reducer was PC-1030, a polycarboxylate-based high-efficiency water reducing agent manufactured by Suzhou Xingbang Chemical Building Materials Co., Ltd. (Suzhou, China). Figure 1 illustrates some of the materials utilized in the experiment.

#### 2.1.2. Uniaxial Compression Tests

**Figure 2:**
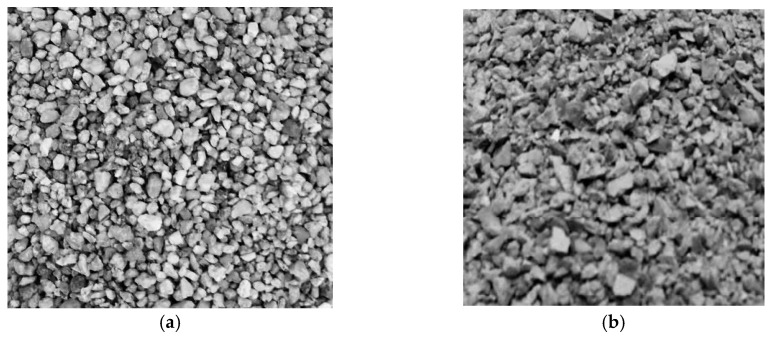
Comparison of fine aggregate particle morphology: (**a**) Natural fine aggregate; (**b**) Recycled fine aggregate.

Concrete with a standard cylinder compressive strength value of 40 MPa is used. Coarse aggregates with a maximum particle size of 15 mm were used. Two types of fine aggregates were selected: recycled fine aggregate and natural fine aggregate. The recycled fine aggregate was obtained by crushing and screening discarded concrete by manual crushing and using a jaw crusher, with a particle size ranging from 0 mm to 5 mm. The natural fine aggregate is comprised of river sand with a fineness modulus of 2.4. Figure 2 illustrates the contrasting particle morphology. It can be seen that the natural sand exhibits a smooth surface and rounded particles, while the recycled fine aggregate appears more angular with a rough surface. The scanning images reveal a substantial presence of original paste within the recycled fine aggregate, resulting in a significant increase in internal pores compared to the natural fine aggregate. This higher pore volume causes the recycled fine aggregate to exhibit a higher water absorption rate, necessitating greater water consumption to achieve saturation in a dry surface condition [21]. The experiment utilized standard Portland cement (grade 42.5). Hooked-end steel fibers with a nominal diameter (df) of 0.65 mm, length (lf) of 25 mm, and length-to-diameter ratio (lf/df) of 38.5 mm are used. These steel fibers possessed a tensile strength of 1060 MPa, according to the suppliers. Tap water served as the mixing water. Table 1 outlines the essential properties of the aggregates utilized, and the test procedures are based on the Chinese standard JGJ 52-2006 [20].

### 2.2. Mix Proportion and Experimental Methodology

#### 2.2.1. Shrinkage Performance Test

The experiment focuses on studying the influence of varying replacement rates (0%, 50%, 100%) of coarse recycled aggregates and different volumes (0%, 0.5%, 1.0%, and 1.5%) of steel fibers on the shrinkage performance of concrete in natural conditions. The fiber content was calculated by taking 0.5%, 1.0%, and 1.5% of the volume of each cubic meter of concrete mixture, then multiplied by the density of the steel, which is 7850 kg/m^3^. First, pour the coarse aggregates and river sand into the mixer and mix for 1.5 min, then add the cement and mix for another 1.5 min, while gradually adding water. After mixing for 3 min, add the steel fibers while continuing to mix for another 3 min. The high-performance polycarboxylate-based water reducer PC-1030 was used, and it was dissolved in the pre-measured water beforehand. A total of 12 mix proportions were designed for the experiment, as outlined in Table 2. According to the specifications [22], each mix proportion group was cast with three compressive strength test specimens measuring 150 mm × 150 mm × 150 mm, as well as shrinkage test specimens measuring 100 mm × 100 mm × 515 mm. The compressive strength test specimens were cured for 28 days under standard curing conditions with a temperature of 20 ± 2 °C and a relative humidity of ≥95% in the door. After drying the surface moisture, the specimens were loaded in a fully automatic pressure testing machine (YAM-2000) produced by Jinan Hengxu Testing Machine Technology Co., Ltd., Jinan, China, according to the Chinese standard GB/T50018-2019 [23], and the specimens were loaded at a rate of 0.5 MPa/s until failure. The peak load was recorded during this process.

**Figure 3:**
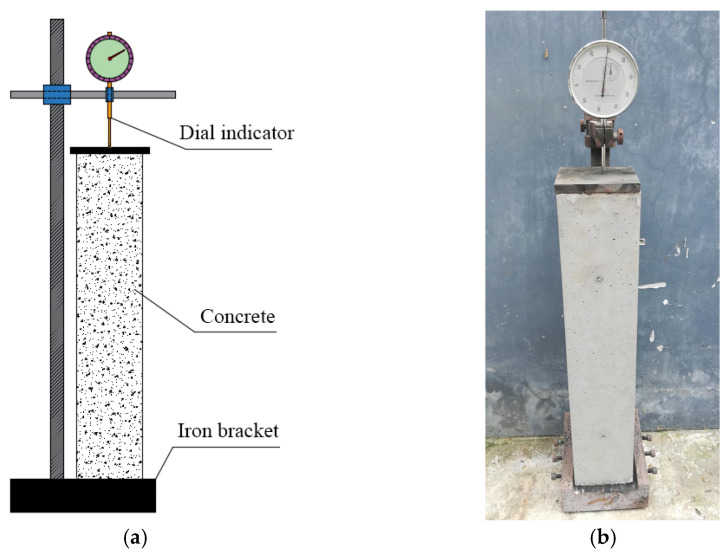
Shrinkage measuring device: (**a**) Instrument diagram; (**b**) Test photo.

The shrinkage specimens were first cured with molds for one day and then placed on the shrinkage measurement instrument illustrated in Figure 3 after demolding. The measurement of concrete shrinkage was conducted using a shrinkage comparator of model BC-160, produced by China’s Beijing Zhongke Lanjian Instrument Equipment Co., Ltd. (Beijing, China). Test procedures are as follows [22]. First, the entire structure was positioned in a cool, windless indoor place, and the initial length was recorded. Subsequently, the lengths of each specimen were measured at 1 d, 3 d, 7 d, 14 d, and 28 d intervals. By subtracting the corresponding age length from the initial length, the shrinkage value of each specimen was determined. During the testing period, the local city experienced an average temperature ranging from 17 °C to 26 °C, with an average relative humidity of 63%.

#### 2.2.2. Uniaxial Compression Tests

According to Chinese Code [24], it is recommended to use a lower sand ratio and not exceed a 50% replacement rate of recycled fine aggregate when designing the reference concrete mix proportions. In this study, the designed sand ratio is 0.35, and the research parameters include the replacement rate of recycled fine aggregate (0%, 30%, 50%, 100%) and the volume fraction of steel fiber (0%, 0.6%, 1.2%, 1.8%). The experiment consists of 10 groups with 3 specimens each, resulting in a total of 30 prism specimens with dimensions of 150 mm × 150 mm × 300 mm. The detailed mix proportions and specimen numbers for each group are provided in Table 3.

Uniaxial compression tests on steel fiber-recycled fine aggregate concrete were conducted by a 5000 kN testing machine. Deformation measurements were made with resistance strain gauges. To prevent strain gauge failure caused by specimen cracking, displacement transducers were symmetrically installed on both sides of the specimen in addition to surface-mounted strain gauges, enabling indirect measurement of vertical deformations. Initial preloading was performed for physical alignment, with a load of approximately 40% of the failure load and a loading rate of 0.5 MPa/s to 0.8 MPa/s. During the main loading phase, displacement control was employed at a rate of 0.1 mm/min [25]. Experimental data were collected automatically by the DH3816 data acquisition system.

## 3. Results and Discussion

### 3.1. Shrinkage Performance Analysis

#### 3.1.1. Test Results and Analysis of Compressive Strength

**Figure 4:**
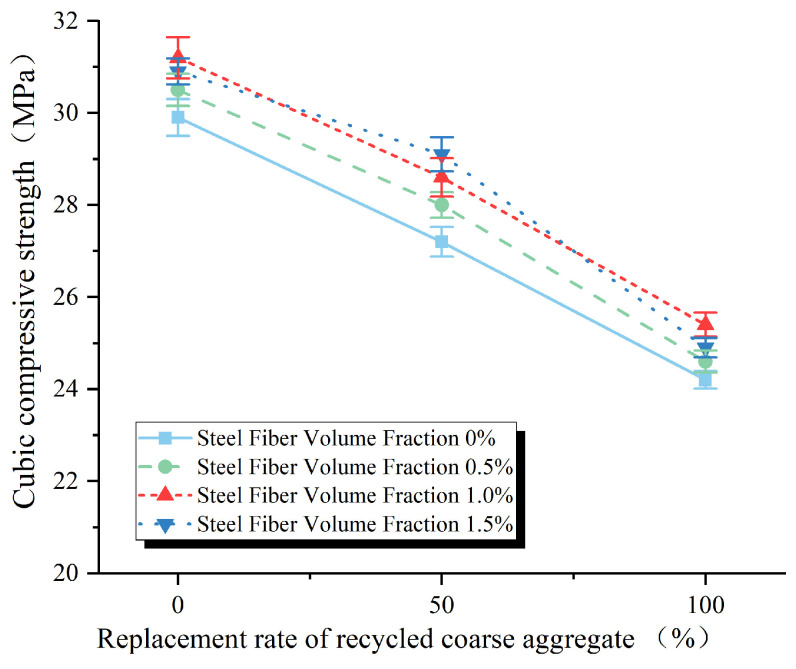
Effect of replacement rate of reclaimed coarse aggregate on compressive strength of cube.

It can be seen in Figure 4 that partially or fully replacing natural coarse aggregate with recycled coarse aggregate will result in a decrease in the compressive strength of concrete cubes. The cube compressive strength test value is shown in Table 4. Statistical findings reveal that the average compressive strength of concrete cubes utilizing 100% recycled coarse aggregate is reduced by 19.1% compared to those composed of pure natural coarse aggregate.

Possible reasons could be attributed to two aspects: Firstly, as the skeletal framework for concrete compression, recycled coarse aggregates inherently possess numerous micro-cracks, with a crushing index about twice that of natural coarse aggregates (as can be seen in Section 2.1.1), thereby resulting in lower strength. Secondly, a significant deposition of aged cement mortar on the surface of recycled coarse aggregates leads to a relatively frail junction between old and new cement mortars.

**Figure 5:**
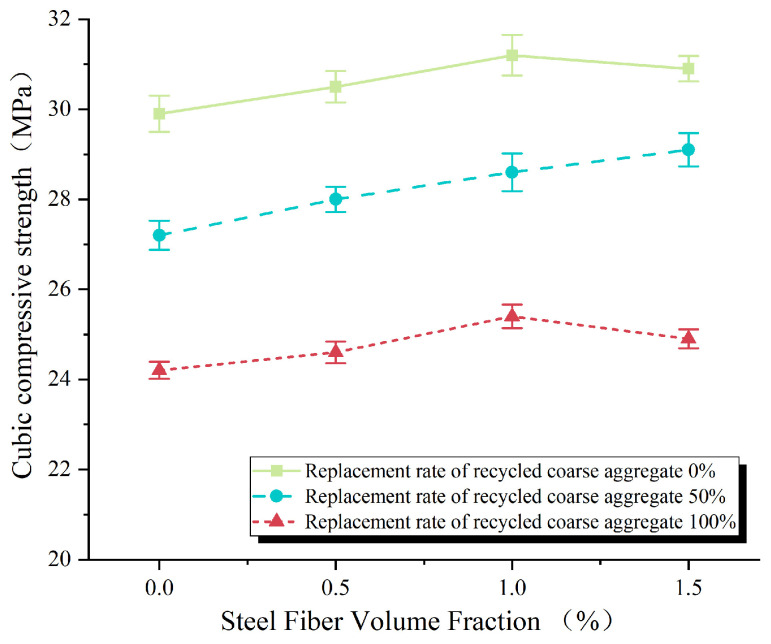
Effect of volume content of steel fiber on compressive strength of cube.

As depicted in Figure 5, when the replacement rate of recycled coarse aggregates reaches 50%, the compressive strength of recycled concrete exhibits an increasing trend alongside the volumetric dosage of steel fibers. The maximum improvement of 6.98% is attained at a steel fiber dosage of 1.5%. Conversely, in cases where the replacement rate of recycled coarse aggregates is 0% or 100%, the compressive strength of recycled concrete initially rises, then declines as the volumetric dosage of steel fibers increases. The peak enhancements achieved are 4.35% and 4.96%, respectively, at a steel fiber dosage of 1.0%.

The ability of steel fibers to enhance the compressive strength of concrete can be attributed to two factors. Firstly, from an energy standpoint, the three-dimensional random distribution of steel fibers within the concrete allows them to absorb a portion of the applied external forces due to their inherent stiffness. Secondly, from a force transmission perspective, the presence of steel fibers acts as a bridging mechanism, restraining the transverse expansion of the concrete under compression, preventing damage, and ultimately improving the load-bearing capacity. When the replacement rates of recycled coarse aggregates are between 0% and 100%, a steel fiber dosage of 1.5% leads to a decrease in the compressive strength of the concrete compared to a dosage of 1.0%. This decrease can be attributed to the agglomeration of steel fibers during the mixing process, resulting in a reduction in the compactness of the concrete and consequently causing a decline in compressive strength.

#### 3.1.2. Test Results and Analysis of Shrinkage

Experimental values of concrete shrinkage rate at different ages under natural conditions are listed in Table 5. 28 day shrinkage and the age-shrinkage curve are detailed studied.

##### 28 Day Shrinkage

**Figure 6:**
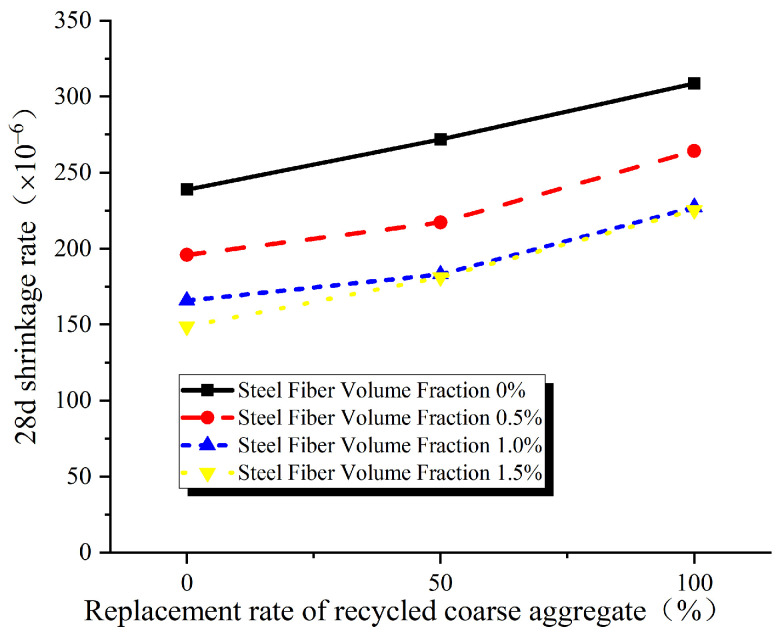
Influence of replacement rate of recycled coarse aggregate on 28 day shrinkage.

As shown in Figure 6, the 28-day shrinkage increases with replacement rates of recycled coarse aggregates increase. Statistical analysis reveals that, compared to concrete without any recycled coarse aggregates, the average 28-day shrinkage increases by 13.86% and 36.85% for concrete with 50% and 100% replacement, respectively. This indicates that the addition of recycled coarse aggregates results in a deterioration of concrete’s shrinkage performance, with a greater degree of deterioration at higher replacement rates.

The primary reasons for this phenomenon can be attributed to two factors. Firstly, there is waste cement adhered to recycled coarse aggregates in mortar, resulting in higher porosity and water absorption. Consequently, a larger amount of water is required during the preparation of recycled concrete, leading to increased free water content and more pronounced shrinkage; this is consistent with the conclusion in the other studies [26,27,28]. Secondly, recycled coarse aggregates possess more internal defects and lower strength, which diminishes their ability to restrain the shrinkage of cement mortar compared to natural aggregates serving as the concrete’s framework. There is also research revealing [14] that as the replacement rate of recycled coarse aggregates increases, the number of pores increases, thereby raising the porosity and pathways for water loss in recycled concrete. Consequently, this intensifies the effects of carbonation shrinkage and drying shrinkage.

**Figure 7:**
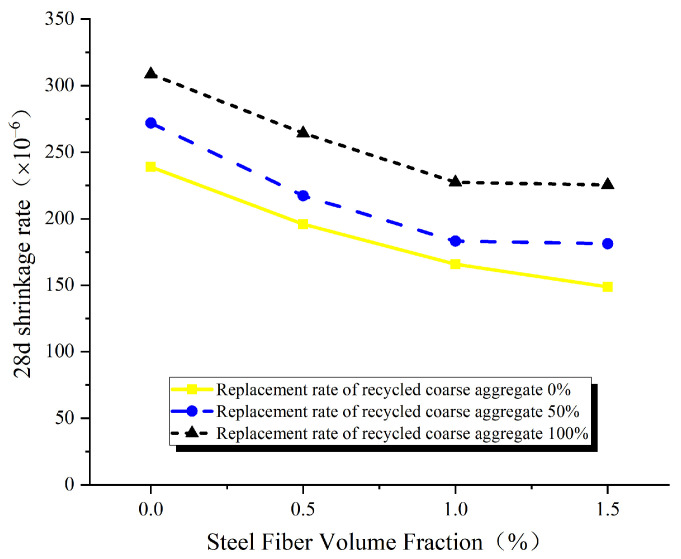
Influence of steel fiber volume fraction on 28 day shrinkage.

As shown in Figure 7, the 28-day shrinkage of concrete is observed to decrease with an increase in the volume fraction of steel fibers. However, when the volume fraction of steel fibers exceeds 1.0% and reaches 1.5%, the 28-day shrinkage rate of concrete shows only a slight reduction or remains relatively stable. This suggests that steel fibers can effectively inhibit concrete shrinkage. However, as the volume fraction of steel fibers increases beyond a certain threshold, their inhibitory effect starts to decline, which is consistent with the findings in the references [15,29,30]. This is attributed to the fact that the presence of uniformly distributed steel fibers forms a complex three-dimensional network within the concrete, effectively inhibiting aggregate settling, enhancing concrete uniformity, and reducing inherent defects. Furthermore, it acts as a barrier to prevent moisture overflow and minimize moisture loss. However, excessive steel fibers complicate the compaction process, leading to increased porosity and an uneven distribution of fibers, thereby weakening their ability to control shrinkage.

##### Age-Shrinkage Curve

**Figure 8:**
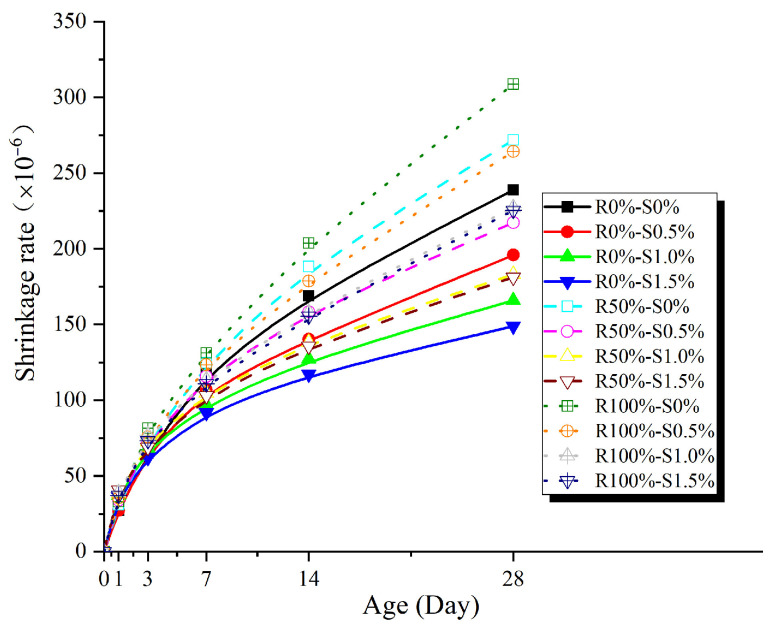
Age-shrinkage curve.

According to Figure 8, the shrinkage rate of all specimens increases with age. However, the rate of increase gradually slows down as the specimens age. During the initial stages, the shrinkage rate exhibits a rapid increase. Furthermore, the specimens with different replacement ratios of recycled coarse aggregates and volume fractions of steel fibers show minor variations in shrinkage rate. This can be attributed to the higher relative humidity within the concrete during this period, which facilitates cement hydration reactions, with chemical shrinkage playing a predominant role. In the later stages, the shrinkage rate of the specimens decreases due to the limited unhydrated cement or the loss of free water, hindering further cement hydration and resulting in a weakening of chemical shrinkage. Additionally, drying shrinkage is closely linked to the internal relative humidity of the concrete. As the internal relative humidity gradually decreases, the rate of drying shrinkage slows down.

#### 3.1.3. Empirical Model of Shrinkage

Figure 6 illustrates that the age-shrinkage curve aligns with the trend of the function *y* = ln(*x* + 1). By considering the correction factors for the replacement ratio of recycled coarse aggregates and the volume fraction of steel fibers, the empirical shrinkage model for steel fiber-reinforced recycled concrete can be derived as follows:

(1) $$\varepsilon_{t}=\beta \ln(t+1)$$

(2) $$\beta =F(\gamma_{r},\gamma_{s})$$

where *ε*_t_ represents the shrinkage rate of concrete at an age of *t* days (×10^−6^), *t* represents the age in days, and *β* is a coefficient associated with the replacement ratio of recycled coarse aggregates (*γ*_r_, %) and the volume fraction of steel fibers (*γ*_s_, %). The age-shrinkage data was first fitted using the software Origin 2018 yielding the coefficients *β* for each specimen, along with their corresponding errors and correlation coefficients, as summarized in Table 6.

Through multiple linear regression, the relationship between the coefficients *β* with the replacement ratio (*γ*_r_, %) of recycled coarse aggregates and the volume fraction (*γ*_s_, %) of steel fibers can be determined as follows:

(3) $$\beta =61.727-12.798\gamma_{s}+0.150\gamma_{r}$$

By substituting Equation (3) into Equation (1), the empirical model for the age-dependent shrinkage of steel fiber reinforced recycled concrete can be derived as follows:

(4) $$\varepsilon_{t}=(61.727-12.798\gamma_{s}+0.150\gamma_{r})\cdot \ln(t+1)$$

**Figure 9:**
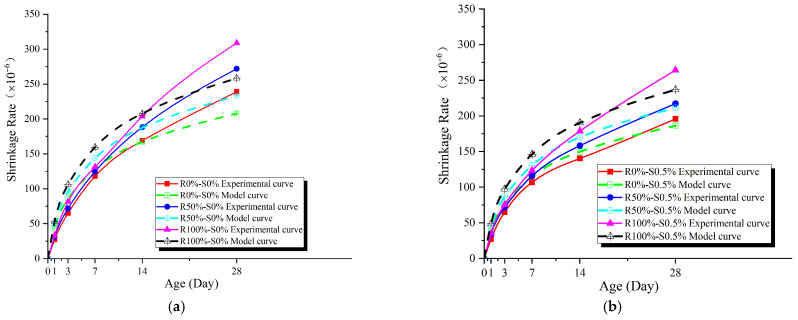

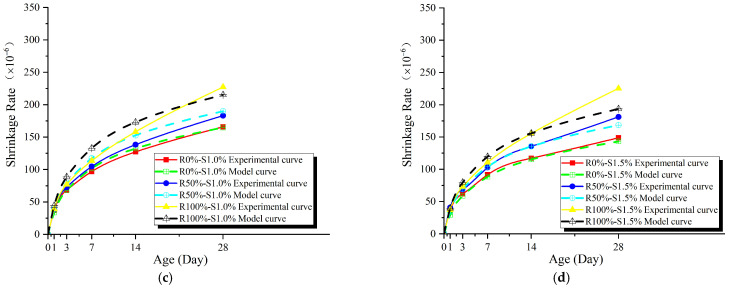
Empirical model validation. (**a**) γ_s_ = 0%; (**b**) γ_s_ = 0.5%; (**c**) γ_s_ = 1%; (**d**) γ_s_ = 1.5%.

Figure 9 illustrates the comparison between the model curve and the experimental curve, demonstrating the high accuracy of the empirical model. 

### 3.2. Constitutive Relationship under Uniaxial Compression

#### 3.2.1. Failure Model

**Figure 10:**
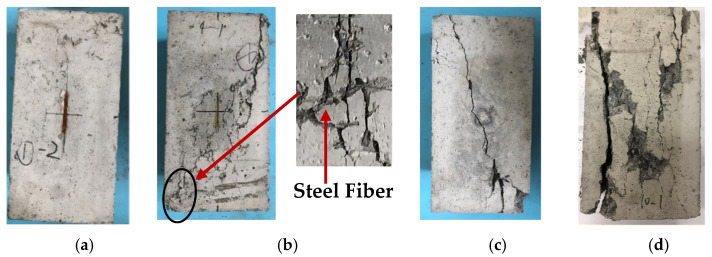
Typical failure modes of some specimens: (**a**) VF1.2-R0; (**b**) VF1.2-R100; (**c**) VF0-R50; (**d**) VF0-R100.

The damaged forms of some specimens are shown in Figure 10. It is observed that the failure patterns of steel fiber-reinforced recycled aggregate concrete are influenced by the replacement ratio of fine aggregates and the volume fraction of steel fibers. The specimens without steel fibers mainly exhibited splitting failure with straight cracks, and the failure surfaces were mostly parallel to the direction of the applied force, resulting in local crushing or spalling of the concrete. After adding steel fibers, the specimens showed shear-type failure, with the failure plane forming an angle with the load axis. Diagonal cracks extended outward with numerous fine cracks. Upon close inspection, steel fibers could be seen at the crack locations. Due to the bridging effect of the steel fibers across the failure surfaces, the cracks were shallower and narrower, maintaining the good integrity of the concrete. Furthermore, a higher replacement ratio of fine aggregates leads to more pronounced crack development. This can be attributed to the fractures occurring primarily at the interfaces between the aggregates and the mortar. Since recycled fine aggregates possess abundant sharp edges, the interface between the new and old cement mortars becomes weaker, resulting in greater levels of interface crack propagation.

#### 3.2.2. Stress-Strain Curve under Uniaxial Compression

**Figure 11:**
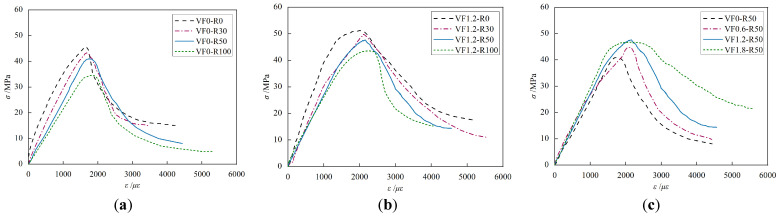
The influence of each parameter on the stress-strain curve: (**a**) Replacement rate of recycled fine aggregate (ordinary concrete); (**b**) Replacement rate of recycled fine aggregate (steel fiber concrete); (**c**) Volume fraction of steel fibers.

To facilitate analysis, the stress-strain curves obtained from three specimens in each group were averaged and presented as the stress-strain curves in Figure 11. Subsequently, various parameters were calculated for different specimens based on their respective stress-strain curves, as summarized in Table 7. Based on the curve variations depicted in the graph, it can be observed that the stress-strain curve of steel fiber-reinforced recycled fine aggregate concrete, shown in Figure 11b, resembles that of regular concrete in Figure 11a. The stress-strain curve characteristics are as follows: Initially, during the loading phase, the concrete behaves elastically with a linear increase in stress. As the load continues to increase, an elastic-plastic stage appears, during which the rate of stress increase is lower than that of strain increase, until it reaches the peak stress. Additionally, it is noticeable from the graph that a higher replacement ratio of recycled fine aggregates leads to a lower slope in the ascending segment and a steeper slope in the descending segment of the stress-strain curve. This is attributed to the increased internal damage and weak interfaces in recycled aggregate concrete, which can result in accelerated crack propagation and increased brittleness of the material. By comparing Figure 11a and Figure 11b, it can be seen that steel fibers can significantly improve the brittleness of recycled concrete, resulting in a reduced slope in the descending segment. An increased volume fraction of steel fibers leads to a smoother decline in the curve. However, in contrast to the replacement ratio of recycled fine aggregates, the volume fraction of steel fibers has a minor influence on the slope of the ascending segment. This is due to the fact that the steel fibers intersect with cracks in the specimens only after the occurrence of longitudinal cracks (i.e., when the curve enters the descending segment), thereby impeding further crack propagation and enhancing the residual strength beyond the peak.

#### 3.2.3. Peak Stress and Peak Strain

**Figure 12:**
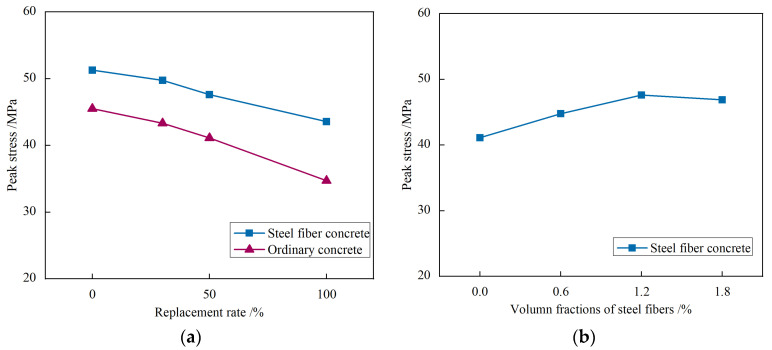
The influence of each parameter on peak stress: (**a**) Replacement rate of recycled fine aggregate; (**b**) Volume fractions of steel fibers.

**Figure 13:**
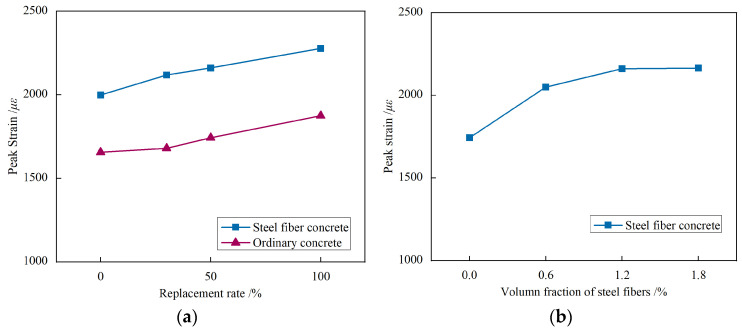
The influence of each parameter on peak strain: (**a**) Replacement rate of recycled fine aggregate; (**b**) Volume fractions of steel fibers.

The influence of each parameter on peak stress and peak strain is shown in Figure 12 and Figure 13. It can be seen that:

(1) Compared to regular concrete (VF0-R0), the peak stress decreases by 4.8%, 9.7%, and 23.7% when the replacement ratio of recycled fine aggregates is 30%, 50%, and 100%, respectively, In the case of steel fiber-reinforced concrete, the peak stress decreases by 3.0%, 7.2%, and 15.1% for replacement ratios of 30%, 50%, and 100% of recycled fine aggregates, respectively, compared to steel fiber-reinforced concrete (VF1.2-R0). As the replacement ratio of recycled fine aggregates increases, the peak strain exhibits an upward trend. This can be attributed to the presence of a considerable amount of cement mortar adhered to the surface of the recycled fine aggregates, which affects cement hydration and increases porosity. Additionally, the lower strength of recycled fine aggregates compared to natural sand leads to increased peak strain and decreased peak stress in recycled fine aggregate concrete. Moreover, the inclusion of steel fibers enables the strength of concrete with a replacement ratio of 100% (VF1.2-R100) to approach that of ordinary concrete VF0-R0 (45.50 MPa). This finding highlights the potential for higher replacement ratios of recycled fine aggregates when steel fibers are incorporated, thereby enhancing their application.
(2) For steel fiber-reinforced recycled aggregate concrete, the peak stress increases by 8.9%, 15.8%, and 14.1%, respectively, when the volume fractions of steel fibers are 0.6%, 1.2%, and 1.8%, compared to ordinary recycled aggregate concrete without steel fibers (VF0-R50). Additionally, as the volume fraction of steel fibers increases, the peak strain significantly rises. This can be attributed to the steel fibers restraining the disintegration of cement mortar and suppressing crack development in the concrete, thereby reducing the scale of micro-cracks and creating a more continuous and uniform stress field [31]. Consequently, the compressive strength of recycled concrete slightly improves, while the peak strain greatly increases. However, it is essential to avoid excessive volume fractions of steel fibers, as it may lead to non-uniform distribution and clustering during the casting process, negatively affecting the workability and compressive strength of the concrete.

#### 3.2.4. Elastic Modulus

The elastic modulus (E) of steel fiber-reinforced recycled aggregate concrete is calculated using the following equation [32]:

(5) $$E=\frac{\Delta \sigma }{\Delta \varepsilon }=\frac{0.4\sigma_{u}-\sigma_{0.5}}{\varepsilon_{0.4\sigma_{u}}-\varepsilon_{0.5}}$$

where *σ*_u_ is the peak stress and unit is MPa, *σ*_0.5_ is the value closest to 0.5 MPa, *ε*_0.4*σ*_ is the strain corresponding to the stress of 0.4*σ*_u_; *ε*_0.5_ is the strain corresponding to the stress of *σ*_0.5_.

**Figure 14:**
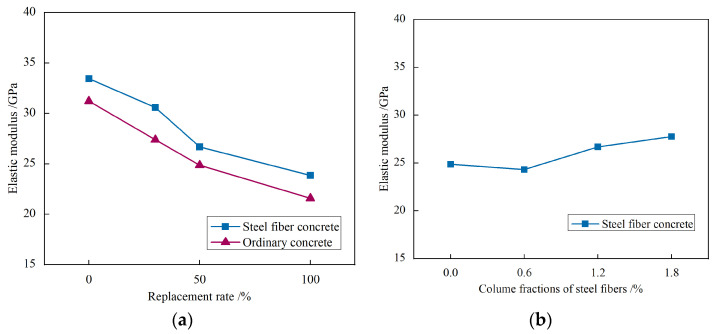
Influence of different parameters on the elastic modulus of steel fiber-recycled fine aggregate concrete: (**a**) Replacement rates of recycled fine aggregate; (**b**) Volume fractions of steel fibers.

The elastic modulus of concrete specimens for each group is presented in Figure 14. The curve in the figure shows that as the replacement ratio of recycled aggregate increases, the elastic modulus of concrete reduces. When the replacement ratio reaches 100%, the elastic modulus decreases by 31%. Since recycled fine aggregate is mainly composed of mortar particles, it has a higher porosity, which weakens the elastic modulus of concrete compared to natural sand. Figure 14b indicates that the variation in the elastic modulus of concrete is insignificant among the different volume fractions of steel fibers in each group. This is due to minimal crack formation in concrete and the limited impact of steel fibers.

#### 3.2.5. Constitutive Model under Axial Compression

**Figure 15:**
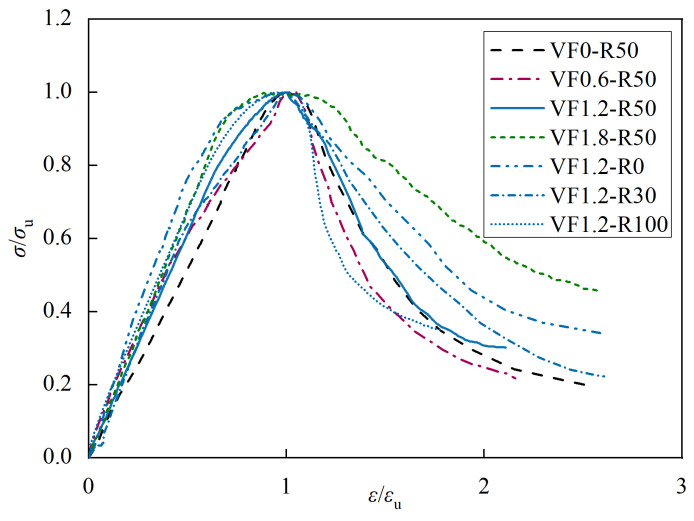
Normalized stress-strain curves for each group.

The stress-strain curves of concrete obtained under different replacement ratios and fiber contents in this experiment are normalized for ease of analysis. The x-axis represents ε/ε_u_ (normalized strain), and the y-axis represents σ/σ_u_ (normalized stress), where σ_u_ and ε_u_ are the peak stress and strain of the specimens, respectively. Figure 15 illustrates the normalized stress-strain curves for each group. The curves of the steel fiber-reinforced recycled aggregate concrete shown in Figure 15 share a similar shape with those of ordinary concrete. Referring to relevant literature on recycled aggregate concrete and steel fiber-reinforced concrete, the following equations can be employed for initial fitting purposes [33,34]:

(6) $$y=\left\{ \begin{array}{l} ax+(3-2a)x^{2}+(a-2)x^{3}\;\;\;\;(0\leq x<1)\\ \frac{x}{b{\left(x-1\right)}^{2}+x}\;\;\;\;\;\;\;\;\;\;\;\;\;\;\;\;\;\;\;\;\;\;\;\;\;\;\;\;\;\;\;\;\;\;\;\;\;(x\geq 1) \end{array}\right.$$

where parameter *a* represents the ratio of the modulus to the peak secant modulus, while parameter *b* reflects the plasticity of steel fiber-reinforced recycled aggregate concrete. A smaller value of parameter *b* leads to a gentler decrease in the curve’s descending segment.

The experimental data was fitted to obtain the values of parameters *a* and *b* for each group (Table 8). The results indicate a good agreement between the experimental points and the fitted curve. Increasing the volume fraction of steel fibers leads to a higher value of parameter *a*, as the fibers inhibit crack development and decrease the peak secant modulus. Conversely, parameter *b* decreases, indicating that the presence of steel fibers moderates the descending segment of the curve by constraining cement mortar disintegration. Moreover, as the replacement ratio of recycled aggregate increases, parameter *a* in the ascending segment generally decreases due to increased porosity and microcracks, resulting in a lower initial elastic modulus of the concrete. In contrast, parameter *b* in the descending segment increases. This is attributed to the higher presence of microcracks and impurities in recycled aggregate, which affect cement hydration and reduce the interfacial bond between concrete phases. Higher replacement ratios lead to faster crack propagation, increased brittleness, and a steeper descending segment. A statistical regression analysis was conducted on the experimental data obtained in this study to establish the relationship between parameters *a*, and *b*, the characteristic parameters of the replacement ratio of recycled aggregate *R*, and the steel fiber content *λ*_f_. *λ*_f_ is defined as *λ*_f_ = *V*_f_ *l*_f_/*d*_f_, where *V*_f_ represents the volume fraction of steel fibers, *l*_f_ is the length of steel fibers, and *d*_f_ is the diameter of steel fibers. The relationship is expressed as follows:

(7) $$\left\{ \begin{array}{l} a=(-0.80\lambda_{f}+3.40)R^{2}+(2.38\lambda_{f}-4.48)R+1.86\\ b=(-22.07\lambda_{f}+12.78)R+(-3.19\lambda_{f}+5.50) \end{array}\right.$$

Equations (6) and (7) form the constitutive equations for the axial compression behavior of steel fiber-reinforced recycled aggregate concrete. When *R* = 0, it degenerates into ordinary concrete without steel fibers. When *λ_f_* = 0, it is applicable to recycled aggregate concrete without the inclusion of steel fibers.

**Figure 16:**
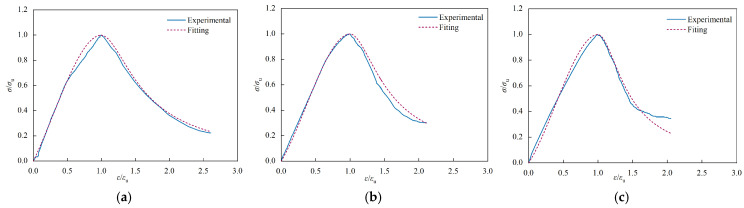
Comparison of the test curve in this paper and the fitting curve: (**a**) VF1.2-R30; (**b**) VF1.2-R50; (**c**) V0-R30.

**Figure 17:**
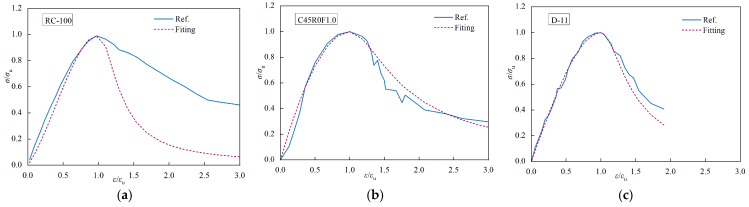
Comparison of test curves in other papers and fitting curves: (**a**) C45R0F1.0 comparison [34]; (**b**) RC-100 comparison [17]; (**c**) D-11comparison [35].

By substituting the calculated values of the parameters obtained from Equation (7) into Equation (6), the normalized stress-strain test curves of steel fiber-reinforced recycled aggregate concrete were compared with those of the computational model. Figure 16 illustrates three representative comparisons of various groups. Therefore, the segmented function demonstrates accuracy in representing the complete stress-strain curve of steel fiber-reinforced recycled aggregate concrete under uniaxial compression. In order to validate the formula, additional data from other references was collected [17,34,35]. Setting *λ_f_* = 0 in Equation (7) when recycled aggregate concrete without the inclusion of steel fibers was used and setting *R* = 0 in Equation (7) when ordinary concrete without steel fibers was used. The comparisons are shown in Figure 17. It can be observed that our model has a better fit for calculating the stress-strain relationship of steel fiber-reinforced conventional concrete but shows higher errors in the descending phase when calculating the stress-strain relationship of recycled aggregate concrete [34]. Considering the limited data on recycled fine aggregate samples in the literature, further analysis focused on polypropylene fiber-reinforced recycled aggregate concrete was also conducted using the experimental results [35]. Although a different fiber type was used, polypropylene fibers can also enhance the mechanical properties of recycled aggregate concrete, providing a useful reference for our study. The comparative results, as shown in Figure 17c, indicate a reasonable fit in the descending phase, demonstrating the good applicability of the proposed constitutive equation in this study. Further research on the constitutive relationship of steel fiber-reinforced recycled aggregate concrete is necessary in the future.

## 4. Conclusions

This paper presents an in-depth investigation into the mechanical performance of steel-fiber-reinforced recycled aggregate concrete. The shrinkage performance of steel fiber concrete with recycled coarse aggregate is first studied, followed by the effects of varying the recycled aggregate replacement rate and steel fiber volume percentage on key properties such as the stress-strain curve, peak stress, peak strain, and elastic modulus of steel-fiber-reinforced concrete with recycled fine aggregate.

As for the shrinkage performance analysis, results show that the shrinkage of steel fiber-reinforced recycled concrete increases with age, but the growth rate slows down, with a similar trend as ordinary concrete. The shrinkage value of recycled coarse concrete at the same age increases with the replacement rate of recycled coarse aggregates, while it decreases with the volume fraction of steel fibers. In addition, an empirical model for the shrinkage of steel fiber-reinforced recycled concrete based on age has been developed, which exhibits a high level of agreement with experimental data.

Through axial load tests on prism specimens, the study also elucidates the influence of varying parameters on key properties such as the stress-strain curve, peak stress, peak strain, and elastic modulus of steel-fiber-reinforced recycled fine aggregate concrete. A constitutive model for steel fiber reinforced recycled aggregate concrete was developed, referring to the existing model and taking the influence of both steel fibers and recycled aggregate into account. The model parameters were determined by fitting the corresponding ascending and descending segments. The fitting results obtained from this model demonstrate good agreement with the experimental data in this paper. Additionally, comparison with test curves from various literatures further confirms the applicability of the proposed model in this study.

This study provides a theoretical basis for characterizing the mechanical performance of steel-fiber-reinforced recycled aggregate concrete, thereby laying the foundation for its future application. However, more experiments are needed to refine our constitutive curves based on mathematical statistics theory [36], and further research is required to investigate the service life of our concrete structures [37].

## Figures and Tables

**Table 1:** Basic physical properties of aggregate.

Aggregate Types	Apparent Density/(kg/m^3^)	Bulk Density/(kg/m^3^)	Crushing Value/%	Water Absorption Rate/%
Natural coarse aggregate	2710	1735	4.5	0.42
Natural fine aggregate	2683	1412	13.2	2.23
Recycled fine aggregate	2786	1470	15.4	5.30

**Table 2:** Mix proportion design of concrete (kg/m^3^).

Number	Cement	River Sand	Coarse Aggregate		Water	Steel Fiber	Water Reducer
			Natural	Recycled			
R0%-S0%	260	750	1220	0	170	0	0
R0%-S0.5%	260	750	1220	0	170	39.3	2.51
R0%-S1.0%	260	750	1220	0	170	78.5	4.32
R0%-S1.5%	260	750	1220	0	170	117.8	6.15
R50%-S0%	260	750	610	610	182	0	0
R50%-S0.5%	260	750	610	610	182	39.3	2.51
R50%-S1.0%	260	750	610	610	182	78.5	4.32
R50%-S1.5%	260	750	610	610	182	117.8	6.15
R100%-S0%	260	750	0	1220	194	0	0
R100%-S0.5%	260	750	0	1220	194	39.3	2.51
R100%-S1.0%	260	750	0	1220	194	78.5	4.32
R100%-S1.5%	260	750	0	1220	194	117.8	6.15

**Table 3:** Grouping of specimens and proportion of steel fiber recycled fine aggregate concrete.

Number	Rate of Recycled Concrete /%	Volume Fraction of Steel Fibers/%	Cement /(kg/m^3^)	Coarse Aggregate /(kg/m^3^)	Natural Fine Aggregate /(kg/m^3^)	Recycled Fine Aggregate /(kg/m^3^)	Water /(kg/m^3^)	Fly Ash /(kg/m^3^)	Steel Fibers /(kg/m^3^)	Water Reducer /(kg/m^3^)
VF1.2-R0	0	1.2	382	810	436	0	196	164	93.6	1.9
VF1.2-R30	30	1.2	382	810	305	131	196	164	93.6	1.9
VF1.2-R50	50	1.2	382	810	218	218	196	164	93.6	1.9
VF1.2-R100	100	1.2	382	810	0	436	196	164	93.6	1.9
VF0-R50	50	0	382	810	218	218	196	164	0	1.9
VF0.6-R50	50	0.6	382	810	218	218	196	164	46.8	1.9
VF1.8-R50	50	1.8	382	810	218	218	196	164	140.4	1.9
VF0-R0	0	0	382	810	436	0	196	164	0	1.9
VF0-R30	30	0	382	810	305	131	196	164	0	1.9
VF0-R100	100	0	382	810	0	436	196	164	0	1.9

Note: In the specimen numbering, VF1.2-R50 denotes concrete with a 50% fine aggregate replacement rate and a 1.2% volume fraction of steel fibers used as recycled materials.

**Table 4:** Cube compressive strength test value.

Number	*f*_cu_ (MPa)	Number	*f*_cu_ (MPa)	Number	*f*_cu_ (MPa)
R0%-S0%	29.9	R50%-S0%	27.2	R100%-S0%	24.2
R0%-S0.5%	30.5	R50%-S0.5%	28.0	R100%-S0.5%	24.6
R0%-S1.0%	31.2	R50%-S1.0%	28.6	R100%-S1.0%	25.4
R0%-S1.5%	30.9	R50%-S1.5%	29.1	R100%-S1.5%	24.9

**Table 5:** Experimental value of concrete shrinkage under natural conditions.

Number	Shrinkage Rate at Different Ages/×10^−6^				
	1 Day	3 Day	7 Day	14 Day	28 Day
R0%-S0%	27.2	65.2	118	169	238.9
R0%-S0.5%	27.2	65.2	107	140.3	195.9
R0%-S1.0%	37.2	68.2	97	127.3	165.9
R0%-S1.5%	36.2	62.2	91.8	117.3	148.9
R50%-S0%	31.1	71.9	124.7	188.4	271.9
R50%-S0.5%	34	71.8	115.8	158.3	217.3
R50%-S1.0%	38.5	72.7	104.7	138.4	183.1
R50%-S1.5%	40.8	68.8	102.9	135.7	181.2
R100%-S0%	33	81.5	131.2	203.8	308.7
R100%-S0.5%	35.5	75.7	123.4	178.8	264.3
R100%-S1.0%	39.1	77.1	113.6	158.3	227.4
R100%-S1.5%	37.2	73.2	110.8	155.5	225.4

**Table 6:** The value of the fitted coefficient *β* and the correlation coefficient *R*^2^.

Number	Coefficient *β*	Error	*R* ^2^
R0%-S0%	63.65407	±3.592	0.95988
R0%-S0.5%	53.99255	±1.901	0.9828
R0%-S1.0%	48.24704	±0.627	0.99726
R0%-S1.5%	44.14647	±0.543	0.99747
R50%-S0%	71.11829	±4.568	0.94925
R50%-S0.5%	60.01522	±2.019	0.98409
R50%-S1.0%	52.62441	±0.785	0.99649
R50%-S1.5%	51.78516	±0.984	0.99428
R100%-S0%	78.74716	±5.802	0.93519
R100%-S0.5%	69.30818	±4.005	0.95711

**Table 7:** Stress-strain curve data.

Number	Fine Aggregate Rate/%	Volume Fraction of Steel Fibers/%	Peak Stress/MPa	Variance	Elastic Modulus/GPa	Variance	Peak Strain/*με*	Variance
VF1.2-R0	0	1.2	51.29	2.02	33.44	2.75	1999	9986
VF1.2-R30	30	1.2	49.76	8.74	30.59	6.78	2119	4981
VF1.2-R50	50	1.2	47.61	5.34	26.68	11.20	2162	6626
VF1.2-R100	100	1.2	43.56	4.18	23.85	5.52	2278	5643
VF0-R50	50	0	41.11	14.56	24.86	5.09	1743	5766
VF0.6-R50	50	0.6	44.77	6.55	24.32	3.44	2050	3915
VF1.8-R50	50	1.8	46.89	13.02	27.76	6.86	2165	6144
VF0-R0	0	0	45.50	15.65	31.21	3.30	1657	5013
VF0-R30	30	0	43.33	9.68	27.39	4.62	1681	2251
VF0-R100	100	0	34.71	8.40	21.59	2.28	1875	4817

**Table 8:** Fitting results of parameters *a* and *b*.

Number	Parameter *a*	Calculated Value	Experiment/ Calculation	Correlation Coefficient *R*^2^	Parameter *b*	Calculated Value	Experiment/ Calculation	Correlation Coefficient *R*^2^
VF1.2-R0	1.84	1.86	0.99	0.9964	2.45	2.20	1.11	0.9914
VF1.2-R30	1.12	1.12	1.00	0.9899	3.61	3.30	1.09	0.9959
VF1.2-R50	1.00	0.93	1.08	0.9984	5.22	4.04	1.29	0.9938
VF1.2-R100	1.49	1.51	0.99	0.9977	7.60	6.87	1.29	0.8872
VF0-R50	0.37	0.47	0.79	0.9703	5.45	7.55	0.72	0.9960
VF0.6-R50	0.79	0.70	1.13	0.9667	6.00	5.97	1.04	0.9830
VF1.8-R50	1.40	1.16	1.21	0.9925	1.76	2.28	0.77	0.9965
VF0-R0	1.86	1.86	1.00	0.9711	4.74	4.19	1.13	0.8320
VF0-R30	0.93	0.82	1.13	0.9843	5.78	6.21	0.93	0.9447
VF0-R100	0.79	0.78	1.01	0.9979	10.81	10.91	0.99	0.9754

## Data Availability

The experimental data used to support the findings of this study are included within the article.
